# *BRAF* V600E-Negative Hairy Cell Leukaemia

**DOI:** 10.1155/2013/513049

**Published:** 2013-04-09

**Authors:** Stephen E. Langabeer, David O'Brien, Anthony M. McElligott, Michelle Lavin, Paul V. Browne

**Affiliations:** ^1^Cancer Molecular Diagnostics, Central Pathology Laboratory, St. James's Hospital, Dublin 8, Ireland; ^2^Department of Haematology, St. James's Hospital, Dublin, Ireland; ^3^Department of Haematology, Trinity College Dublin, Dublin, Ireland

## Abstract

Since the initial report of the *BRAF* V600E mutation in hairy cell leukemia, numerous investigators have demonstrated the presence of this activating mutation in nearly all cases of this disease. A case of hairy cell leukemia is documented with a classical clinical, morphological, immunophenotypic, and cytochemical profile in which the *BRAF* V600E was not detected. The diagnostic and therapeutic implications are discussed.

## 1. Introduction

Acquired mutations of *BRAF* have been described in several tumour types and while *BRAF* mutations undoubtedly contribute to malignant proliferative processes, the heterogeneity of tumour types implicates further cell lineage-specific pathogenic events. Since the initial report of the *BRAF* V600E mutation in hairy cell leukemia (HCL) [1], this mutation has been sought in numerous cohorts, the majority of which confirm the presence of the *BRAF* V600E in classical HCL and its absence in HCL variant and other mature B-cell malignancies, therefore, providing a rationale for targeted therapy [2]. We have previously identified a single case of HCL in which the *BRAF* V600E was not detected and in which further studies were limited by the lack of suitable material [3]. This patient subsequently relapsed allowing fuller investigation. The clinical course is described with diagnostic and therapeutic implications discussed.

## 2. Case Report

**Figure 1:**
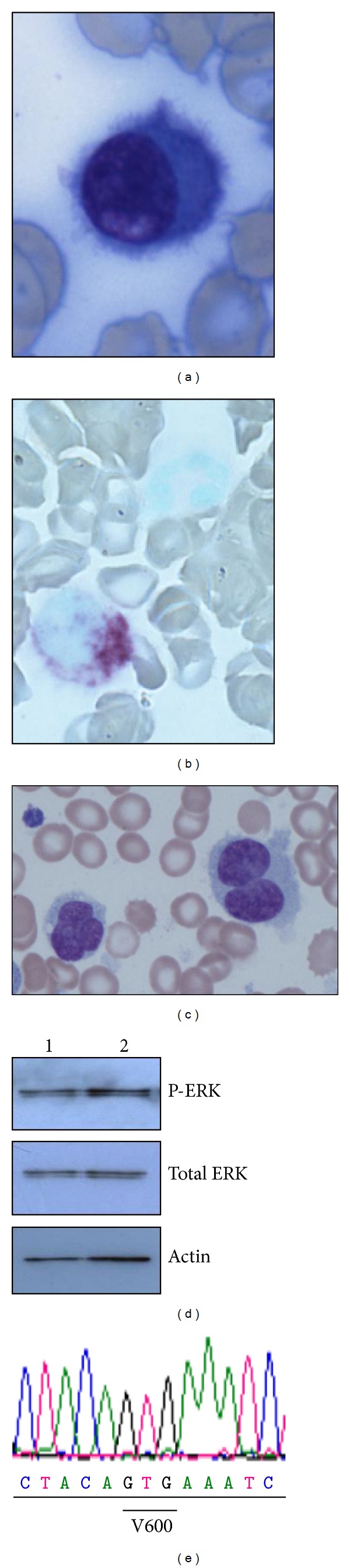
(a) Hairy cell morphology at second relapse; (b) TRAP staining of hairy cells at second relapse; (c) multinucleate hairy cells at third relapse; (d) Western blot demonstrating no differences in phospho-ERK levels between untreated (lane 1) and PLX4720 treated (lane 2) cells; (e) wild-type sequence of patient *BRAF *exon 15.

A 32-year-old male presented in 1981 with severe pancytopenia and a macular haemorrhage. He was noted to have splenomegaly and a bone marrow infiltration consistent with a lymphoproliferative disorder. A splenectomy was performed in October 1981 with subsequent resolution of his pancytopenia. The splenic sample confirmed the diagnosis of HCL. In April 1982, he presented with back pain with initial investigations suggesting osteoporosis with vertebral collapse. A bone biopsy confirmed involvement of the dorsal spine with HCL. At this time his peripheral counts were normal. He commenced and completed six cycles of chlorambucil 20 mg daily orally for three days a month in May 1982 with the back pain responding well. He remained in remission until an increasing leucopenia in October 2009 suggested a further relapse, confirmed by bone marrow aspirate and biopsy. In the bone marrow, kappa-restricted hairy cells represented 13% of B lymphocytes (Figure 1(a)), were tartrate resistant acid phosphatase-positive (Figure 1(b)), and possessed the classical HCL immunophenotype (CD5−, CD10−, CD11c+, CD19+, CD20+, CD22+, CD23−, CD25+, CD79b−, CD103+, CD200+, FMC7+). Allele-specific PCR did not detect the *BRAF *V600E mutation [3]. He commenced on a five-day course of subcutaneous cladribine in November 2009 with both clinical and immunophenotypic remissions achieved. Neutropenia signalled another relapse in June 2012. The bone marrow hairy cells (39% of B lymphocytes) had the same immunophenotype as the previous relapse, several of which were multinucleate, possibly a treatment related effect (Figure 1(c)). The presence of the *BRAF* V600E mutation was again not detected by allele-specific PCR and a further two commercial assays (Competitive Allele-Specific Taqman PCR, Applied Biosystems, Paisley, UK, and Cobas, Roche, Burgess Hill, UK). The B-cell population was further enriched by CD20 magnetic bead selection: treatment of these B-cells with the BRAF inhibitor PLX4720 showed no decrease in ERK phosphorylation (Figure 1(d)) while Sanger sequencing of *BRAF* exons 11 and 15 revealed wild-type sequence (Figure 1(e)). He recommenced cladribine subcutaneously for five days with eight weekly doses of rituximab and remained clinically well at last followup. 

## 3. Discussion

Acknowledging that variations in immunophenotype and immunohistochemical staining patterns often occur in HCL [4, 5], the features of the patient described herein, taken together with the molecular characterisation, lead us to conclude that this case represents *BRAF* V600E-negative HCL. Sequencing and the more sensitive allele-specific PCR approaches failed to detect the mutation while demonstration of the lack of phospho-ERK response to inhibitor, a valid surrogate of the *BRAF* V600E [6, 7], corroborates the lack of RAF/MEK/ERK pathway activation. While the vast majority of HCL patients genotyped harbour the *BRAF* V600E, a significant minority of cases of mutation-negative cases have also been documented [8–10]. If targeted therapy for HCL is to be realised [11–13], then genotyping for *BRAF* V600E must be a prerequisite.
